# Prevalence and determinants of decision regret in long-term prostate cancer survivors following radical prostatectomy

**DOI:** 10.1186/s12894-023-01311-9

**Published:** 2023-08-23

**Authors:** Lukas Lunger, Valentin H. Meissner, Benedikt C. G. Kopp, Andreas Dinkel, Stefan Schiele, Donna P. Ankerst, Jürgen E. Gschwend, Kathleen Herkommer

**Affiliations:** 1 Department of Urology, Klinikum rechts der Isar, School of Medicine, Technical University of Munich, Ismaninger Strasse 22, 81675 Munich, Germany; 2 Department of Psychosomatic Medicine and Psychotherapy, Klinikum rechts der Isar, School of Medicine, Technical University of Munich, Munich, Germany; 3 Departments of Mathematics and Life Science Systems, Munich Data Science Institute, Technical University of Munich, Garching, Germany

**Keywords:** Decision regret, Radical prostatectomy, PSA-anxiety, Prostate cancer-related anxiety, Depression

## Abstract

**Background:**

Patients with localized prostate cancer (PC) are faced with a wide spectrum of therapeutic options at initial diagnosis. Following radical prostatectomy (RP), PC patients may experience regret regarding their initial choice of treatment, especially when oncological and functional outcomes are poor. Impacts of psychosocial factors on decision regret, especially after long-term follow-up, are not well understood. This study aimed to investigate the prevalence and determinants of decision regret in long-term PC survivors following RP.

**Methods:**

3408 PC survivors (mean age 78.8 years, SD = 6.5) from the multicenter German Familial PC Database returned questionnaires after an average of 16.5 (SD = 3.8) years following RP. The outcome of decision regret concerning the initial choice of RP was assessed with one item from the Decision Regret Scale. Health-related quality of life (HRQoL), PC-anxiety, PSA-anxiety, as well as anxiety and depressive symptoms were considered for independent association with decision regret via multivariable logistic regression.

**Results:**

10.9% (373/3408) of PC survivors reported decision regret. Organ-confined disease at RP (OR 1.39, 95%CI 1.02–1.91), biochemical recurrence (OR 1.34, 1.00-1.80), low HRQoL (OR 1.69,1.28–2.24), depressive symptoms (OR 2.32, 1.52–3.53), and prevalent PSA anxiety (OR 1.88,1.17–3.01) were significantly associated with increased risk of decision regret. Shared decision-making reduced the odds of decision regret by 40% (OR 0.59, 0.41–0.86).

**Conclusions:**

PC survivors may experience decision regret even after 16 years following RP. Promoting shared decision-making in light of both established and novel, potentially less invasive treatments at initial diagnosis may help mitigate long-term regret. Awareness regarding patients showing depressive symptoms or PSA anxiety should be encouraged to identify patients at risk of decision regret in need of additional psychological support.

## Background

Patients with localized prostate cancer (PC) are faced with a variety of therapeutic options, ranging from active surveillance to radical prostatectomy (RP), external beam radiation or focal therapies. Selecting the most suitable treatment can be difficult and requires the treating physician to spend adequate time and effort to involve patients during the decision-making process. To ensure patients make informed and subsequent satisfactory decisions regarding the most suitable primary treatment, clinicians ought to take into account tumor-specific characteristics, life expectancy, possible complications and side effects, concomitant diseases, and last but not least, expectations of the individual patient [1].

Notably, up to a quarter of PC patients may experience significant regret regarding their initial treatment choice [2–8]. Available data suggests that patients may experience decision regret not only during the early postoperative period [2, 3, 9], but also beyond five years following primary treatment [4, 5]. A recent longitudinal study from our group including 1003 PC survivors showed that decision regret does not naturally resolve, but remains stable or increases slightly even 20 years after RP [10]. Previous studies have shown poor post-treatment oncological and functional outcomes, early health-related quality of life (HRQoL) declines, and PC-related anxiety to be associated with later decision regret [2, 3, 5, 9]. Similarly, previous studies suggested that depression [11] and a patient’s passive role during decision-making can be associated with later decision regret, too [3, 4, 6–8, 12, 13], and that shared-decision making may help reduce decision regret [14].

Studies on decision regret to date have typically had small sample sizes and short follow-up. They have primarily focused on decision regret associations with oncological or surgery-related functional parameters, such as urinary continence, erectile function, and bowel symptoms, rather than psychosocial factors [3–5, 8].

A more holistic understanding of decision regret and its determinants would (a) increase awareness among treating clinicians regarding available preventive strategies during the initial diagnosis consultation and (b) facilitate the identification of patients suffering from regret and associated risk factors in need of additional psychological support during the course of survivorship. Despite the negative implications of significant regret on long-term psychosocial and physical health following RP, unawareness regarding the presence, associated risk factors, and strategies to prevent decision regret is common.

This study investigates the prevalence of decision regret in a large cohort of long-term PC survivors following primary RP, comprising to our knowledge, the largest number of cases with complete follow-up data > 15 years. It additionally quantifies the association of sociodemographic, clinicopathological, and psychological characteristics with decision regret in order to ultimately improve informed provider-patient shared decision-making regarding RP.

## Methods

### Study population and procedures

The prospective German Familial Prostate Cancer Database was initiated in 1993 and, to date, includes > 36,000 patients with PC and their relatives. Since 1993, patients with newly diagnosed PC have been recruited and annually surveyed, independent of their family history. The database is updated annually via questionnaires including sociodemographic, clinicopathological, and psychosocial characteristics. Individual informed consent was obtained from all participants; the study was approved by the ethical review committee of the Technical University of Munich. All methods were performed in accordance with relevant guidelines and the Declaration of Helsinki. Detailed database descriptions were previously provided [15, 16].

Patients were eligible for this study if they (1) had RP as first-line treatment and (2) returned the decision regret questionnaire between November 2021 and January 2022.

### Measures

Sociodemographic features recorded included age at survey (years), level of education (low, intermediate, high or tertiary), children (yes vs. no), and partnership status (yes vs. no).

Clinicopathological characteristics included years since RP, positive PC family history (defined as at least one first-degree relative with PC), presence of secondary cancer, prostate-specific antigen (PSA) level at diagnosis (ng/ml), organ-confined disease at RP (≤ pT2c and pN0), presence of lymph node or distant metastases at RP (pN status/M status), presence of biochemical recurrence (defined as rising PSA value ≥ 0.2 ng/ml since RP), and current therapy (androgen deprivation therapy, chemotherapy, radiation therapy vs. none).

Decision regret was assessed using one item *(“Would go for the same choice if you had to do it over again (yes vs. no)”*) from the Decision Regret Scale (DRS), based on the highest item-total correlation published in a previous study [17].

Decision-making roles regarding primary PC treatment (RP vs. alternative PC treatments) were retrospectively assessed using the validated self-report Control Preference Scale (CPS) [18–20]. Based on five statements, three decision-making roles were distinguished: active (patient makes the final decision alone or after considering the physician’s opinion), shared (the patient and physician make the final decision together) or passive (the physician makes the final decision alone or after considering the patient’s opinion).

Health-related quality of life (HRQoL) was assessed using items 29 and 30 of the EORTC QLQ-C30 [21]. Patients were considered to have a good HRQoL with a score ≥ 70 following previously published cut-off values [22].

Cancer-related anxiety (PC/PSA anxiety) was assessed using the Memorial Anxiety Scale for Prostate Cancer (MAX-PC) (modified PC anxiety subscale with 4 of the original 11 items and all 3 items of the PSA anxiety subscale [23–25]). Patients were considered to have PC/PSA anxiety when patients agreed with at least 1 item per subscale, respectively.

General anxiety and depressive symptoms were assessed using the Patient Health Questionnaire-4 (PHQ-4), an ultra-brief screening tool consisting of a two-item depression scale (PHQ-2) and a two-item anxiety scale (Generalized Anxiety Disorder 2 (GAD-2)), with a cut-off score ≥ 3 suggestive of clinical levels of depression and anxiety [15, 26].

### Statistical analyses

Data analyses were conducted using the Statistical Analysis System (SAS), version 9.4 (SAS Institute Inc., Cary, NC, USA). To analyze differences in sociodemographic, clinicopathological, and psychological characteristics by the presence of decision regret, chi-square-, Wilcoxon-, and t-tests were performed as indicated. Multivariable logistic regression analyses were performed to determine the association of selected sociodemographic, clinicopathological, and psychological characteristics with decision regret. Odds ratios (OR), 95% confidence intervals (CI) and p-values were reported, with the 0.05 level considered statistically significant.

## Results

### Characteristics of the study population

Out of 5797 participants contacted, 3408 (58.7%) returned the questionnaires, 2076 were lost to follow-up and 200 had died. A total of 113 patients did not reply to the decision regret questionnaire and were therefore not included in this analysis (detailed non-responder analysis not shown). There was no statistically significant difference in age (p = 0.079), positive PC family history (p = 0.9666) or screening for depression/anxiety (p = 0.281 and 0.111, respectively) between responders and non-responders. Non-responders had a statistically significant lower quality of life (p = 0.004), had more frequently non-organ-confined tumor (p = 0.009), slightly lower educational levels (p = 0.044), and more often reported passive decision-making regarding surgery (0.035).

Among the 3408 study participants, the mean age was M = 78.8 (SD = 6.5) years and survey completion occurred after a mean of M = 16.5 (SD = 3.8) years following RP (Table 1). Overall, 10.9% of patients reported current decision regret based on the single-item DRS related to the initial RP treatment choice. Most patients (61.9%) reported to have made a shared decision, whereas 27.0% had taken an active and 11.1% a passive role regarding their initial treatment choice. The median HRQoL score was Md = 75 (IQR = 25). A total of 8.6% and 7.1% of patients screened positive for depression or anxiety, respectively.

**Table 1:** Patient characteristics (N = 3,408)

	n	%
**Sociodemographic characteristics**		
Age at survey, mean (SD), y	78.8	6.5
Level of education		
Low	1179	38.9
Intermediate	537	17.7
High	377	12.4
Tertiary	940	31.0
Partnership	2635	86.6
Children	3010	88.7
**Clinicopathological parameters**		
Years since RP, mean (SD), y	16.5	3.8
Positive PC family history	1364	40.0
Secondary cancer	416	12.2
PSA at diagnosis, mean (SD), ng/ml	7.2	6.0
Organ defined disease at RP	2420	71.5
Lymph node invasion at RP	133	3.9
Distant Metastasis at RP	1	0.03
Biochemical recurrence	1239	36.4
Current therapy	368	10.8
Androgen deprivation therapy	364	98.9
Chemotherapy	3	0.8
Radiation therapy	5	1.3
**Psychological parameters and HRQoL**		
Decision regret		
Yes	373	10.9
No	3035	89.1
Treatment decision making		
Active	912	27.0
Shared	2094	61.9
Passive	376	11.1
HRQoL, mean (SD)	69.5	19.3
PC anxiety, mean (SD)	2.6	3.0
PSA anxiety, mean (SD)	0.4	1.1
Depression (PHQ), mean (SD)	1.0	1.2
Anxiety (GAD-2), mean (SD)	0.8	1.1

* Numbers reflect sample sizes (n (%)) except when reported otherwise

***Abbreviations***: RP, radical prostatectomy; PSA, prostate-specific antigen; PC, prostate cancer; BCR, biochemical recurrence; HRQoL, Health-related quality of life. Y, years.

Overall, there was no difference in the frequency of decision regret across sociodemographic characteristics (Table 2). Decision regret was significantly more frequent in patients reporting biochemical recurrence as compared to patients who had no biochemical recurrence (13.3% vs. 9.6%, respectively, p < 0.001, Table 2).

**Table 2:** Presence and distribution of decision regret across sociodemographic, clinicopathological, and psychological characteristics*

	*Decision regret*				
	Yes		No		
	n	%	n	%	p-value
	373	10.9	3035	89.1	
**Sociodemographic characteristics**					
Age at survey, mean (SD), y	78.9 (6.3)		78.8 (6.5)		0.712
Level of education					0.196
Low	113	9.6	1066	90.5	
Intermediate	64	11.9	473	88.1	
High	49	13.0	328	87.0	
Tertiary	96	10.2	844	89.8	
Partnership					0.222
Yes	277	10.5	2358	89.5	
No	51	12.5	356	87.5	
Children					0.744
Yes	331	11.0	2679	89.0	
No	40	10.4	343	89.6	
**Clinicopathological characteristics**					
Years since RP, mean (SD)	16.8 (3.61)		16.4 (3.80)		0.080
Positive PC family history					**0.015**
Yes	171	12.5	1193	87.5	
No	202	9.9	1842	90.1	
Secondary cancer					0.274
Yes	39	9.4	377	90.6	
No	334	11.2	2658	88.8	
PSA at diagnosis, mean (SD), ng/ml	9.7 (10.1)		10.6 (12.7)		0.893
Organ defined disease at RP					0.134
Yes	279	11.5	2141	88.5	
No	94	9.7	871	90.3	
Lymph node invasion at RP					0.469
Yes	12	9.0	121	91.0	
No	360	11.0	2906	89.0	
Biochemical recurrence					**< 0.001**
Yes	165	13.3	1074	86.7	
No	208	9.6	1961	90.4	
Current active therapy					0.123
Yes	49	13.3	319	86.7	
No	324	10.7	2716	89.3	
**Psychological characteristics and HRQoL**					
Decision making					**0.005**
Active	107	11.7	805	88.3	
Shared	204	9.7	1890	90.3	
Passive	57	15.2	319	84.8	
HRQoL					**< 0.001**
< 70	236	14.2	1430	85.8	
≥ 70	129	7.6	1561	92.4	
PC anxiety					**< 0.001**
Yes	153	14.1	933	85.9	
No	190	8.9	1937	91.1	
PSA anxiety					**< 0.001**
Yes	44	22.1	155	77.9	
No	308	10.0	2766	90.0	
Positive screening for clinical depression					**< 0.001**
Yes	63	22.7	214	77.3	
No	277	9.4	2663	90.6	
Positive screening for clinical anxiety					**< 0.001**
Yes	43	18.9	185	81.1	
No	296	10.0	2675	90.0	

*Numbers reflect sample sizes (n (%)) except when reported otherwise.

***Abbreviations***: RP = radical prostatectomy; PSA = prostate specific antigen; PC = prostate cancer; HRQoL = health-related quality of life; SD = standard deviation; IQR = interquartile range.

Decision regret was most frequent in patients reporting passive (15.2%) and active decision-making (11.7%), while among patients reporting shared decision-making, regret was least common (9.7%) (p = 0.005, Table 2). Furthermore, decision regret was more frequent in patients screening positive for depression (22.7% vs. 9.4%, p < 0.001), anxiety (18.9% vs. 10.0%, p < 0.001), low HRQoL (14.2% vs. 7.6% p < 0.001), prevalent PC anxiety (14.1% vs. 8.9%, p < 0.001), and prevalent PSA anxiety (22.1% vs. 10.0%, p < 0.001).

### Multivariable logistic regression analysis

**Fig. 1:**
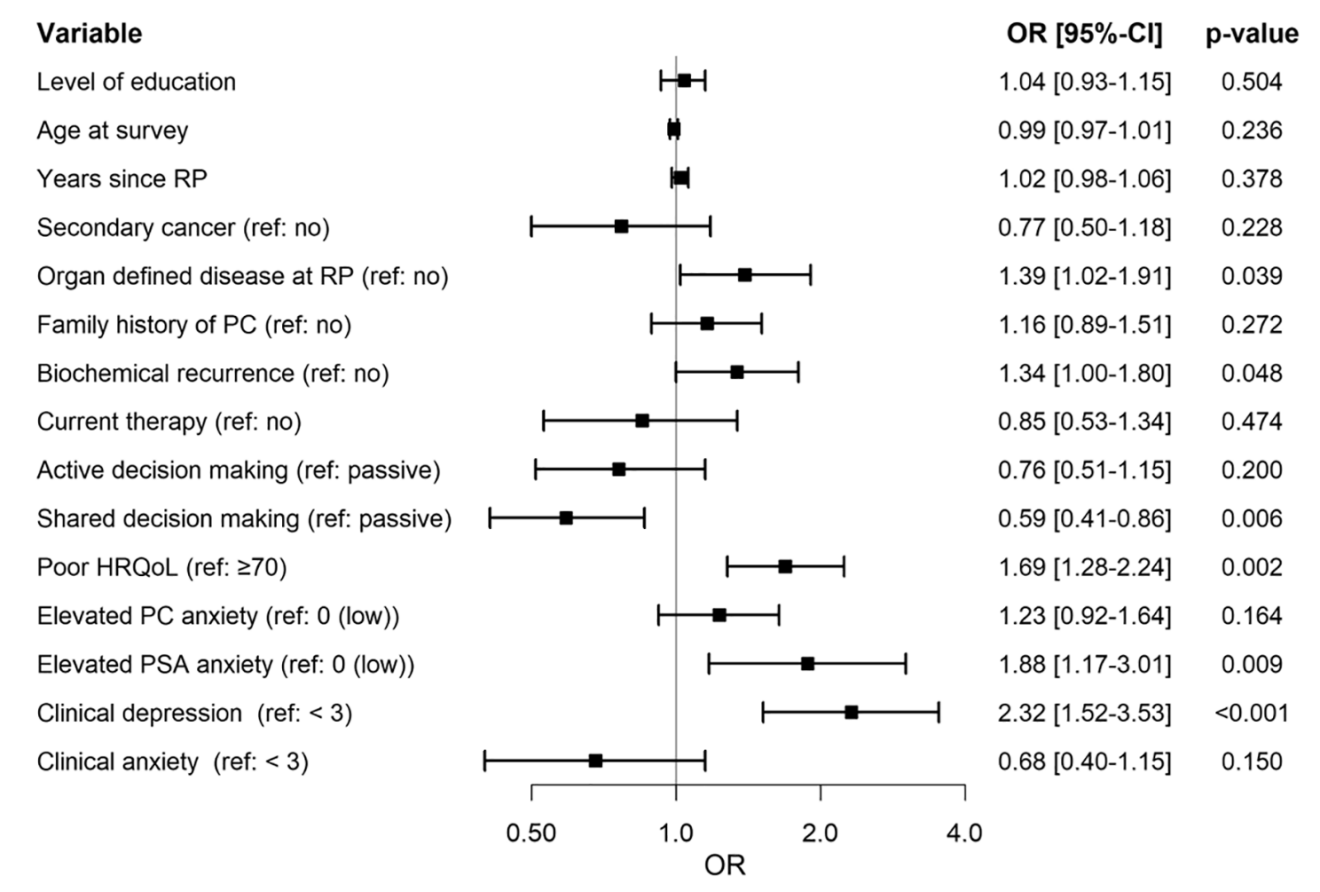
Forest plot to illustrate multivariable logistic regression analyses carried out to determine the association of selected sociodemographic, clinicopathological, and psychological characteristics with decision regret **Abbreviations**: RP = radical prostatectomy. HRQoL = Health related quality of life. PC = prostate cancer. PSA = prostate specific antigen

Multivariable analysis (see Fig. 1) revealed that organ-confined disease (≤ pT2c and pN0) at RP (OR 1.39, 95%CI (1.02–1.91), biochemical recurrence (OR 1.34 95%CI (1.00-1.80)), and low HRQoL (OR 1.69, 95%CI (1.28–2.24)) were significantly associated with decision regret (Fig. 1). Shared decision-making was associated with lower odds of decision regret (OR 0.59, 95%CI (0.41–0.86)). Positive screening for depression (OR 2.32, 95%CI (1.52–3.53)) and prevalent PSA anxiety (OR 1.88, 95%CI (1.17–3.01), but not positive screening for anxiety (OR 0.68 95%CI (0.40–1.15)) or PC anxiety symptoms (OR 1.23, 95% CI (0.92–1.64)) were significantly associated with decision regret.

## Discussion

Following RP for localized PC, survivors may experience regret regarding the initial choice of treatment. The short-term impact of poor functional and oncological outcomes on decision regret is extensively explored; yet, little is known on the prevalence of decision regret and its psychosocial determinants following RP based on long-term follow-up.

Prevalence of decision regret in the current study (10.9%) was lower, yet still comparable to the only other study with a comparable follow-up period of 15 years (15.0%) [4].Of note, that study reported on a small cohort of only 934 patients, while the current study investigated a substantially larger cohort of 3408 PC survivors. Shorter-term studies with follow-up periods < 7 years reported rates of regret affecting up to one quarter of patients following definitive surgical treatment [2–5, 7, 9]. A US study suggested that PC survivors may experience more regret as time passes and certain side effects become more long-lasting or permanent [4]. On the other hand, postoperative complications may have a greater impact on younger men, thus, explaining higher rates of regret in PC survivors during the early to mid-term postoperative phase [27]. Also, older PC survivors may expect a naturally declining functional status or have accommodated to persisting functional impairments, therefore reporting less regret with older age. Here, neither age at survey or time since RP were associated with long-term regret, suggesting that other factors may play a more dominant role surrounding decision regret.

The result of this study that shared decision-making was associated with lower decision regret 16.5 years after primary RP is concordant with previous studies investigating decision regret [3–6, 28]. Facing decisions with potentially substantial impacts on oncological and functional outcomes can be challenging for newly diagnosed PC patients [29]. As different treatments are preference sensitive, patients should be made aware of available options with respective risk and benefit profiles, especially considering the possibility of overtreatment in patients who could benefit from active surveillance [1]. Clearly, an oncological patient consultation should reflect the current state of the art for treatment of PC. Consequently, regret may develop or amplify even long after the primary treatment has ended due to patients becoming aware of novel, potentially less invasive and more tolerable options than the one they had initially chosen. This aspect may have even more importance during long-term follow-up in patients with postoperative low-risk tumors experiencing chronic complications and in whom active surveillance or other novel treatments might have been a viable alternative. Findings of this study support that hypothesis: presence of organ-confined disease (≤ pT2c and pN0) was associated with higher treatment decision regret, suggesting that patients may experience regret as they learn that a less invasive strategy, such as active surveillance, might have been viable. Patients, being non-medical professionals in most cases, may not be able to self-discern pros and cons associated with old versus novel treatment concepts and require professional support to filter new information regarding such aspects. Additionally, some patients may experience difficulties adapting psychologically to a “new life” following primary surgery while others do not. Addressing such emotions and discussing present and especially past treatment landscapes during follow-up may be important to avoid development of self-blame in light of subjectively “better”, novel or less invasive alternatives regarding optimal treatment.

Ideally, the opportunity to discuss available options and complications should arise during the consultation with the treating physician, involving both surgeons and radiotherapists to provide diverse perspectives. Including both treating disciplines can contribute to a more comprehensive and satisfactory decision-making process for the patients. Yet, to tailor information to a patient’s need and encourage shared decision-making, many oncologists lack the time to establish a strong relationship with their patients within the narrow timeframe from diagnosis to treatment discussions. As a consequence, many patients choose to entertain a variety of second opinions regarding their decision, although this may amplify preexisting confusion due to the lack of agreement among different specialists [30]. Moreover, the importance of the internet as a source of reliable, independent information has grown substantially. A recent study reported that although up to 60% of 4636 long-term PC survivors had consulted the internet regarding information on their disease, many patients had difficulties interpreting and trusting the provided information [31]. Although the internet provides patients with an uncomplicated access to information, most sources are unverified and uncontrolled, causing significant anxiety and distress while seeking to make the best treatment decision [30, 32]. In a study in 2011 of 349 patients regarding patient centeredness, decision-making and information preferences, only 7% reported to rely on the treating physician as the sole source of information. Moreover, most patients reported that the internet had similar importance during the decision-making process as the treating physician [33]. In an attempt to address this gap and prevent long-term decision regret, providing reviewed and publicly available, tailored patient information on available treatments and complications could be promoted and emphasized.

In this study, regret was more common in patients with biochemical recurrence, low HRQoL, cancer-related anxiety (PC and PSA anxiety), and patients screening positive for depressive and anxiety symptoms. On multivariable logistic regression, biochemical recurrence, low HRQoL, positive screening for depression and PSA anxiety (by single item cut-off) were associated with regret. Regarding the influence of PC-related anxieties (PC/PSA anxiety) on regret, available data are ambiguous: some investigations indicate a clear association between PSA anxiety and regret [4] while others do not [10]. Also, the results typically rely on continuous scaling, rendering the interpretation and comparison of available findings to the current study and between one another difficult. However, the results of this current study, based on single-item cut-offs for PC-related anxieties, support that patients with elevated PSA anxiety were more likely to experience significant long-term decision regret, which is in line with results of a previous study [4]. PSA relapse was similarly associated with regret on multivariable regression, congruent with prior findings [6], highlighting that both psychosocial and clinicopathological parameters are relevant determinants of decision regret.

Taken together, the current study is the largest cross-sectional cohort analysis of PC survivors to assess the prevalence and determinants of decision regret over the longest reported mean follow-up period of 16 years to date. The results underline the importance of both preventive and diagnostic tools to address this adverse outcome of decision regret following primary treatment.

Some limitations are noteworthy. First, functional parameters were not included in this study; it is well described that postoperative dysfunctions (sexual or urinary) may have an impact on long-term regret [4, 7, 34, 35]. However, as organ dysfunctions are naturally more frequent in older men, it remains unclear to what extent these factors impact long-term decision regret in a population with a mean age of 78.8. Also, the focus of this study was to rather understand and investigate the associations of psychological parameters with regret, rather than investigate the well-established relationship of organ dysfunction on regret. Second, the responses to the questionnaires were subject to recall-bias, as patients had to remember circumstances of a decision made roughly 16 years ago. Third, the investigated, large cohort includes only patients following RP; conclusions may therefore be one-sided and conclusions regarding other treatment modalities can therefore not be made.

## Conclusions

Taken together, the current analysis confirms the considerable prevalence of decision regret based on a single-item DRS in a large cohort of PC survivors even after many years following primary therapy. Shared decision-making was associated with less regret, whereas low HRQoL, positive screening for depression, and PSA anxiety were associated with more decision regret in this study. To prevent and/or reduce decision regret in PC survivors, early intervention via patient education on all available treatments via shared decision-making should be promoted. This may be particularly important when choosing between definitive treatment versus active surveillance as an option.

## Data Availability

The datasets used and/or analysed during the current study are available from the corresponding author on reasonable request.

## List of abbreviations

CI: Confidence interval
CPS: Control Preference Scale
DRS: Decision regret scale
GAD: Generalized Anxiety Disorder
HRQoL: Health-related quality of life
IQR: Interquartile range
M: Mean
OR: Odds ratio
PC: Prostate cancer
PHQ: Patient Health Questionnaire
PSA: Prostate specific antigen
RP: Radical prostatectomy
SD: Standard deviation
